# Survival rate and associated factors affecting pulpectomy treatment outcome in primary teeth

**DOI:** 10.1002/cre2.473

**Published:** 2021-07-16

**Authors:** Methaphon Songvejkasem, Prim Auychai, Oitip Chankanka, Siriporn Songsiripradubboon

**Affiliations:** ^1^ Department of Pediatric Dentistry, Faculty of Dentistry Chulalongkorn University Bangkok Thailand; ^2^ Department of Preventive Dentistry, Faculty of Dentistry Prince of Songkla University Songkhla Thailand

**Keywords:** general anesthesia, primary teeth, pulpectomy, survival rate

## Abstract

**Objectives:**

To evaluate the survival rates of pulpectomized primary teeth treated under general anesthesia (GA) or local anesthesia (LA), and to determine which factors affected tooth survival following pulpectomy.

**Materials and methods:**

This retrospective study collected data from dental records. Patients under 5 years of age received dental treatment under GA or LA during 2007–2016, with at least one anterior or posterior tooth receiving a pulpectomy, were recruited. Pulpectomy was considered a failure if the tooth required extraction or retreatment due to pulp treatment failure. Survival analysis was used to assess the outcome. The cumulative survival probability was analyzed with the Kaplan–Meier estimator. Cox regression analysis was used to evaluate the associations between tooth survival and possible prognosis factors; sex, age, dental arch (upper/lower), tooth type (anterior/posterior), molar type (first/second molar), molar location (upper/lower molar), root filling material type, restoration type, preoperative radiographic findings and presence of pathologic root resorption.

**Results:**

Two hundred and twenty‐seven primary teeth were included. At the 5‐year follow‐up, the survival rates of the pulpectomized teeth treated under GA and LA were 81.4% and 87.4%, respectively, which were not significantly different (*p* ≥ 0.05). A radiolucency on the preoperative radiograph was the only factor associated with tooth extraction or retreatment following pulpectomy, with a hazard ratio of 3.88 (95% CI = 1.29–11.65).

**Conclusions:**

Pulpectomized primary teeth treated under GA and LA demonstrated high survival rates. Preoperative radiolucency is a possible associated factor that decreases tooth survival following pulpectomy.

**Why this paper is important**

- Pulpectomy treatment under GA and LA provided high 5‐year cumulative survival rates, which were not significantly different.
- Pulpectomy treatment in teeth with a preoperative radiolucency were 3.9‐fold as likely to fail as teeth without pathology.
- Based on our findings, practitioners could apply these findings and discuss with caregivers about the treatment options, outcomes, and prognosis of pulpectomized teeth.

## 1 INTRODUCTION

Dental caries is a very common chronic disease in children. Caries can cause pain and infection that affects their quality of life, including normal eating and sleeping; followed by growth and developmental impairment (Abanto et al., 2011; Ayhan et al., 1996). Moreover, teeth with extensive decay may require extraction, leading to early tooth loss (Colak et al., 2013). Along with masticatory function, maintaining aesthetics, and preventing speech and psychological problems, primary teeth preserve space for the permanent teeth and maintain normal succedaneous tooth eruption timing. Therefore, premature primary tooth loss results in the mesial drift of permanent molars, causing malocclusion. To maintain pulpally involved primary teeth in the oral cavity, pulpectomy is the alternative treatment to extraction (American Academy of Pediatric Dentistry, 2019).

Extensive dental caries, which usually involves multiple teeth, and because very young children are uncooperative, may require comprehensive dental treatment in an operating room using general anesthesia (GA) (Schroth et al., 2016). This modality allows the child's dental treatment to be completed in a single visit. Comprehensive dental treatment under GA comprises preventive and restorative procedures, including pulp treatment and tooth extraction (Amin et al., 2016). Many studies have demonstrated the success and survival rates of restorations and vital pulp therapies performed under GA (Al‐Eheideb & Herman, 2003; Blumer et al., 2019; Lin & Lin, 2015), however, the data on long‐term tooth survival following pulpectomy under GA is limited and inconsistent (Amin et al., 2016; Chen et al., 2020; Tang & Xu, 2017). Although pulpectomy conducted in clinical settings, using local anesthesia (LA) has a high success rate (80%–100%) (Coll et al., 1985; Ozalp et al., 2005; Pramila et al., 2016), some medical facilities avoid providing pulpectomy treatment under GA and do not include it as one of their treatment options under GA. Therefore, it would be beneficial to clarify that the survival rates of pulpectomized teeth treated under GA and LA are similar. This information would demonstrate whether pulpectomy treatment under GA is worthwhile. Thus, pulpectomy could be an alternative to extraction for young children with pulpally involved primary teeth treated under GA.

Currently, several clinical studies have reported the potential factors associated with the success and tooth survival after pulpectomy (Amin et al., 2016; Coll et al., 1985; Trairatvorakul & Chunlasikaiwan, 2008). However, these studies were usually conducted using a small sample size, short‐term follow‐up period, and assessed only binary outcomes of success or failure. Few studies have reported an association between the potential clinical factors and survival of primary teeth following pulpectomy, such as tooth position, molar type, and type of restoration (Amin et al., 2016; Chen et al., 2020; Rawson et al., 2019). However, the results are inconsistent, and the association between preoperative radiographic findings and pulpectomy survival has not been determined. Therefore, the aim of this study was to evaluate the survival rate of pulpectomized primary teeth treated under GA or LA, and to determine which clinical and radiographic factors affect primary tooth survival following pulpectomy. These data could help dentists selecting the appropriate treatment for each affected tooth treated under GA.

## 2 METHODS

### 2.1 Study design and participants

This retrospective study was approved by the Ethics Committee of the Faculty of Dentistry, Chulalongkorn University, Bangkok, Thailand (HREC‐DCU. 2018‐118). The data were retrieved from the dental records of children under 5‐year‐olds who had at least one tooth undergo pulpectomy treatment under GA or LA without sedation at the Pediatric Dentistry Clinic, Faculty of Dentistry, Chulalongkorn University between 2007 and 2016. The teeth included in the study met the following inclusion criteria: (1) teeth had received pulpectomy due to carious exposure, (2) pulpectomy was completed in one visit, (3) definitive restorations were completed within 30 days, (4) teeth were treated by pediatric dentistry residents, (5) pre‐operative radiographs were available, and (6) teeth were followed up at least 6 months after treatment. The sample size calculation was based on Hair et al. To estimate the associations between factors and survival outcome using a multiple regression model and achieve 80% power with a significance level of 0.05, a minimum of five events per variable is preferred (Hair et al., 2010). Together with using the survival probability of pulpectomized teeth reported by Tang and Xu (Tang & Xu, 2017), a sample size of 222 teeth was required.

### 2.2 Pulpectomy procedure and follow‐up

Although the teeth that underwent pulpectomy in this study were not treated by a single operator, all teeth were treated and followed up following the institution's protocol as described below.

Prior to treatment, periapical radiographs of the teeth were taken using a radiographic film holder and the bisecting angle technique. After being anesthetized, the tooth was isolated with a rubber dam. An access opening to the pulp chamber was prepared using a sterile high‐speed diamond bur with coolant. Radicular pulp tissue was removed with broaches. The working length of each root was determined using an electronic apex locator (EAL) [RootZX® (J. Morita Co)]. The canals were cleaned and shaped using endodontic K‐files and with constant irrigation with normal saline solution. When the canals were completely prepared, they were dried with sterile paper points and obturated with either zinc oxide eugenol (ZOE) or Vitapex (Neo Dental International, Inc.). IRM® (Dentsply, Caulk) was then placed into the pulp chamber. The tooth was definitively restored with a stainless steel crown, tooth with an unrestorable crown was restored as a coping with either an amalgam, resin composite (Filtek Z350™, 3M ESPE), or glass ionomer restoration (Fuji II LC®, GC Corporation). After pulpectomy, the teeth were followed up periodically until their succedaneous permanent teeth erupted. Clinical assessment was performed at every regular recall visit, which was usually 3–6 months depending on the child's caries risk. Radiographic assessment was performed every 6–12 months, depending on the clinical signs and clinicians' discretion.

### 2.3 Data collection

Electronic database and manual data collection were used to identify the teeth to be included into the study. Teeth treated during 2009–2016 were identified by screening with an electronic dental record program using treatment codes for pulp treatment. Furthermore, the eligibility of the teeth treated before 2009 was determined by manually reviewing the dental records of all pediatric patients treated in these years. Each tooth was labeled with a numerical code. The data was recorded from the patients' dental charts. A pre‐operative radiograph of each tooth was assessed using a fluorescent light box in a darkened room to determine the radiographic findings of each tooth prior to the treatment by a trained, standardized examiner. The factors potentially associated with treated tooth survival were: sex (boy/girl), age at pulpectomy (<36 months/≥36 months), dental arch (maxillary/mandibular), tooth type (anterior/posterior), molar type (first/second molar) and location (upper/lower), type of root canal filling material, type of final restoration, preoperative radiographic findings (no pathology, widened periodontal space and/or discontinuous lamina dura, radiolucency at the periapical area or furcation), and pathologic root resorption (presence/absence). The radiographic interpretation reliability was evaluated by re‐examining 10% of the samples. For the intra‐examiner reliability, the radiographs were interpreted 2 weeks apart. Inter‐examiner agreement and intra‐examiner reliability were calculated using Kappa statistics, which demonstrated an almost perfect agreement of 0.89 and 1.00, respectively.

### 2.4 Treatment outcome

The survival time of the pulpectomized teeth was estimated. The starting point was identified as the pulpectomy date. The study endpoint was set to be at 5‐year observation period. The teeth were followed up until treatment failure or the last date that they were present in the dental records. The teeth were evaluated clinically and radiographically on a regular basis, which were included in the patients' periodic recall. The treatment failure criteria was adapted from Casas et al. (Casas et al., 2004), and pulpectomized primary teeth were categorized into three groups:

1. *N* = no clinical signs or symptoms of infection; pain, gingival swelling, purulence, pathologic mobility, and no evidence of pathologic change in bone or root resorption, except for that associated with exfoliation.
2. *P*_o_ = pathologic radiographic change without clinical signs or symptoms, not requiring immediate extraction.
3. *P*_*x*_ = pathologic radiographic change with clinical signs or symptoms, indicating immediate extraction or retreatment.

In this study, treatment was considered a failure if the tooth was defined as *P*_*x*_, that is, required extraction or retreatment due to pulpectomy failure, and the end of the follow‐up period was noted as the date that the tooth was indicated to be extracted or retreated. If a tooth naturally exfoliated, was lost to follow up, or extracted due to other reasons, it was considered as a censored observation, which indicated that the tooth did not have the interested event (failure) during the time under observation. Censored observations contributed to the total number at risk up to each time point that they ceased to be followed. The end of the follow‐up period for censored cases was defined as the last date that the tooth was present in the dental record. If the tooth was present in the oral cavity without any further treatment until the end of observation, the end of the follow‐up period was defined as the 5‐year follow‐up date. A tooth where the end of observation was reached before the 5‐year follow‐up and showed no event of interest during the observation period, was considered as censored data. The end of the follow‐up period of this tooth was defined as the last date it was present in the dental record.

### 2.5 Statistical analysis

Statistical analysis was performed using STATA 14.1 (StataCorp LLC, College Station, Texas, USA). The Kolmogorov–Smirnov test was used to determine the normal distribution of age between groups. The difference in age between groups was tested using either the independent *t*‐test or Mann–Whitney *U* test, depending on whether or not the data was normally distributed. The difference in the proportions in each categorical variable was compared using the Chi‐square test. The Kaplan–Meier estimator was used to evaluate the cumulative survival probability and median survival time of the pulpectomized teeth treated under each anesthetic type: GA or LA. Each variable was tested with a univariate Cox regression model. A *p* < 0.05 was considered significant. Variables with a *p*‐value ≤0.2 were entered into the final multivariate Cox regression model together with anesthetic type as covariates to determine the influence of each variable and the adjusted hazard ratios were reported. The data from the two anesthetic types were forced in the model to determine the factors affecting pulpectomized tooth survival.

## 3 RESULTS

### 3.1 Descriptive data

There were 272 teeth from 162 patients (78 males and 84 females) recruited into the study, with a mean age of 40.3 ± 8.9 months (range 17–59 months). One hundred and twenty teeth from 57 patients (52.6% male) received a pulpectomy under GA and 152 teeth from 105 patients (45.7% male) were treated under LA. The mean age of the patients receiving a pulpectomy under GA was 34.9 ± 7.3 months, while those who received a pulpectomy under LA had a mean age of 44.7 ± 7.6 months. The age distribution between the GA and LA groups was skewed (*p* < 0.01) and the mean age between the GA and LA groups was significantly different (*p* < 0.001). The distribution of tooth characteristics by anesthetic type is presented in Table 1. Most of the teeth were in the maxillary arch in the GA and LA groups. Upper anterior teeth comprised the majority of the treated teeth (38.2%), followed by lower molars (33.1%), upper molars (21.7%), and lower anterior teeth (7.0%). In the LA group, the proportion of first and second molars was equal. However, the proportion of first molars was slightly higher than second molars in the GA group. Most of the teeth were filled with Vitapex in each group. The proportions of root canal filling materials in each group were significantly different (*p* < 0.001). All posterior teeth were restored with a stainless steel crown as full coverage. Most of the anterior teeth (71.8%) were restored with stainless steel crowns, and the others (28.2%) were restored with amalgam, resin composite or glass ionomer as a coping. The proportions of the restorations in the anterior teeth and other tooth‐level variables between the GA and LA groups were not significantly different (*p* ≥ 0.05).

**TABLE 1:** Distribution of tooth characteristics by anesthetic type

Variables	Treated under GA		Treated under LA		Total		*p‐*value
Tooth							
Dental arch, *n* (%)							0.14	
Maxillary	66 (55.0)		97 (63.8)		163 (59.9)		
Mandibular	54 (45.0)		55 (36.2)		109 (40.1)		
Tooth type, *n* (%)							0.50	
Anterior	57 (47.5)		66 (43.4)		123 (45.2)		
Posterior	63 (52.5)		86 (56.6)		149 (54.8)		
Molar type, *n* (%)							0.21	
First molar	40 (63.5)		43 (50.0)		83 (55.7)		
Second molar	23 (36.5)		43 (50.0)		66 (44.3)		
Location, *n* (%)							0.51	
Upper molar	23 (36.5)		36 (41.9)		59 (39.6)		
Lower molar	40 (63.5)		50 (58.1)		90 (60.4)		
Treatment							
Root canal filling materials, *n* (%)							<0.001*	
Zinc oxide eugenol	18 (15.0)		52 (34.2)		70 (25.7)		
Vitapex	102 (85.0)		100 (65.8)		202 (74.3)		
Final restorations, *n* (%)							0.11	
Stainless steel crown	109 (90.8)		128 (84.2)		237 (87.1)		
Coping	11 (9.2)		24(15.8)		35 (12.9)		
Preoperative radiographic findings							
Pathology, *n* (%)							0.11	
No pathology	42 (35.0)		68 (44.7)		110 (40.4)		
Widened PDL space and/or discontinuity of lamina dura	26 (21.7)		20 (13.2)		46 (16.9)		
Radiolucency at the periapical tissue or furcation	52 (43.3)		64 (42.1)		116 (42.6)		
Pathologic root resorption, *n* (%)							0.67	
No root resorption	116 (96.7)		144 (94.7)		260 (95.6)		
Root resorption	4 (3.3)		8 (5.3)		12 (4.4)		

Abbreviations: GA, general anesthesia; LA, local anesthesia.

*Note*: Significant differences determined using the Chi‐square test.

* *p* < 0.001.

### 3.2 Survival probability of the pulpectomized teeth

**FIGURE 1:**
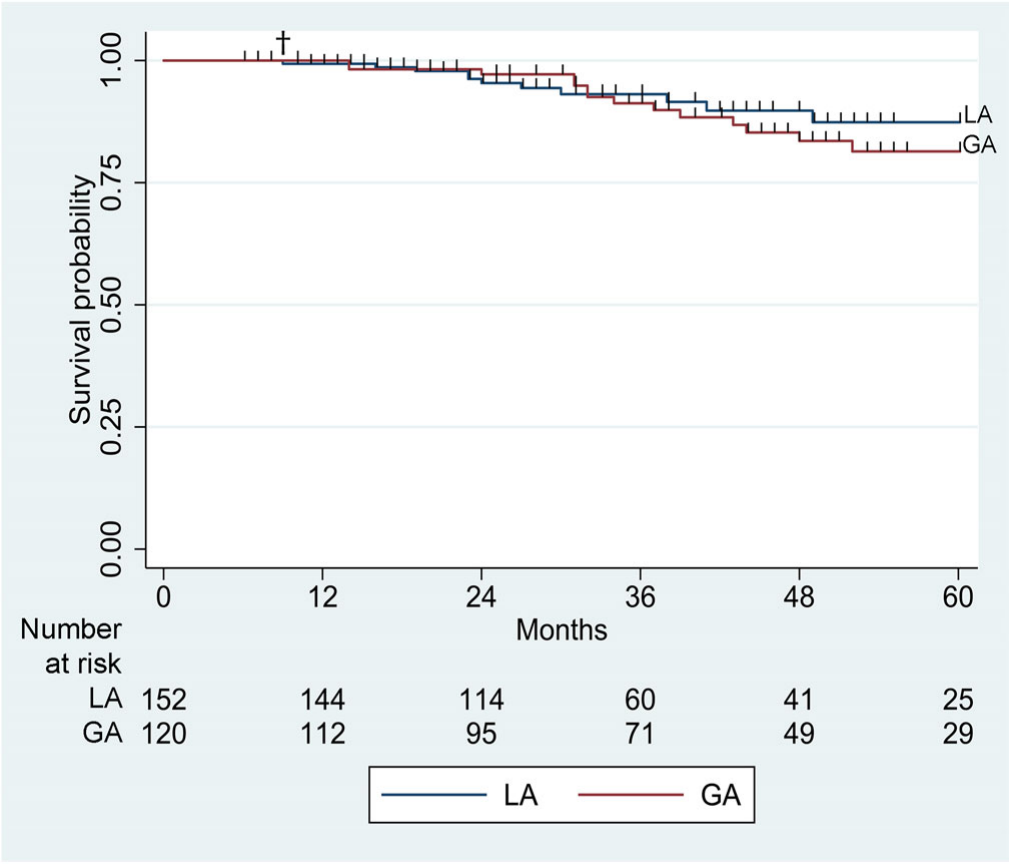
Kaplan–Meier survival curves of the pulpectomized teeth treated under GA (red line) or LA (blue line), † represents censored observations

By the end of the study, 14/120 teeth that received a pulpectomy under GA and 11/152 teeth treated under LA were deemed failures. Most failures occurred within 3 years after the pulpectomy. The 5‐year survival probability of the teeth that received a pulpectomy under GA and LA were 81.4% (95% CI 70.0–88.8) and 87.4% (95% CI 77.0–93.3), respectively. The univariate analysis revealed no significant difference between the survival probabilities of the teeth treated under GA or LA (*p* = 0.56). The median survival time after pulpectomy under both settings was more than the 5‐year follow‐up period. The Kaplan–Meier survival curve of the pulpectomies performed under GA or LA is illustrated in Figure 1.

### 3.3 Association between prognostic factors and pulpectomized tooth survival

Table 2 presents the results of the univariate and multivariate Cox regression analyses on the effect of each prognostic factor on pulpectomized tooth survival. The univariate regression analysis demonstrated that a radiolucency at the periapical tissue or furcation and pathologic root resorption were potentially associated with pulpectomized tooth survival (*p*‐value ≤0.2). Therefore, these two factors were included in the multivariate Cox regression analysis together with anesthetic type as covariates. After controlling for (1) pathologic root resorption and (2) anesthetic type, a preoperative radiolucency at the periapical tissue or furcation demonstrated a significant effect (*p* = 0.02) on tooth survival. In contrast, age, sex, dental arch, tooth type, anesthetic type, type of root canal filling materials, type of final restoration, or the presence of pathologic root resorption were not significantly associated with pulpectomized tooth survival.

**TABLE 2:** Effect of prognostic factors on survival of teeth receiving pulpectomy treatment

Characteristics	*N*	Failures (%)	Median survival (m)	Univariate			Adjusted		
				HR	95% CI	*p*‐value^‡^	HR	95% CI	*p*‐value^‡^
Sex									
Male	145	11 (7.6)	>60	1					
Female	127	14 (11.0)	>60	1.58	0.72, 3.49	0.26	‐	‐	‐
Age									
< 36 months	88	11 (12.5)	>60	1					
≥ 36 months	184	14 (7.6)	>60	0.80	0.36, 1.77	0.58	‐	‐	‐
Dental arch									
Maxillary	163	11 (6.7)	>60	1					
Mandibular	109	14 (12.8)	>60	1.56	0.70, 3.43	0.27	‐	‐	‐
Tooth type									
Anterior	123	11 (8.9)	>60	1					
Posterior	149	14 (9.4)	>60	1.11	0.50, 2.45	0.80	‐	‐	‐
*Molar type*									
First molar	83	6 (7.2)	>60	1					
Second molar	66	8 (12.1)	>60	1.54	0.53, 4.44	0.43	‐	‐	‐
*Location*									
Upper molar	59	5 (8.5)	>60	1					
Lower molar	90	9 (10.0)	>60	0.97	0.33, 2.91	0.96	‐	‐	‐
Anesthetic type									
General anesthesia	120	14 (11.7)	>60	1			1		
Local anesthesia	152	11 (7.2)	>60	1.27	0.27, 2.80	0.56	1.25	0.56, 2.77	0.58
Root canal filling materials									
Zinc oxide eugenol	70	4 (5.7)	>60	1					
Vitapex	202	21 (10.4)	>60	1.83	0.63, 5.33	0.27	‐	‐	‐
Final restorations									
Stainless steel crown	237	20 (8.4)	>60	1					
Coping	35	5 (14.3)	>60	1.78	0.66, 4.79	0.25	‐	‐	‐
Preoperative radiographic findings									
No pathology	110	4 (3.6)	>60	1			1		
Widened PDL space and/or discontinuity of lamina dura	46	4 (8.7)	>60	2.22	0.56, 8.90	0.30	2.16	0.54, 8.68	0.28
Radiolucency at periapical tissue or furcation	116	17 (14.7)	>60	4.14	1.39, 12.31	0.01*	3.88	1.29, 11.65	0.02*
Pathologic root resorption									
No root resorption	260	23 (8.9)	>60	1			1		
Root resorption	12	2 (16.7)	>60	3.68	0.85, 15.87	0.08	2.41	0.55, 10.58	0.24

Abbreviations: 95% CI, 95 percent confidence interval; HR, hazard ratio.

*Note*: Significant differences were determined using the Cox regression analysis, *p < 0.05.

## 4 DISCUSSION

Tooth survival has been used as an outcome measure in other dental treatments, including endodontic treatment, dental implants, and periodontal surgery in permanent teeth (Dannewitz et al., 2006; Ng et al., 2011). However, the survival of primary teeth receiving pulpal treatment has been rarely reported. Most of the studies evaluated the efficacy of pulp treatment in primary teeth based on clinical and radiographic criteria. These criteria represent signs of apical healing. However, these criteria cannot determine the probability that a tooth would be retained in the oral cavity after a pulpectomy. Tooth survival outcome is also a patient‐important outcome, and is additional evidence concerning the advantages of a specific treatment for patients. Therefore, this outcome should be assessed and reported (Smail‐Faugeron et al., 2013; Waterhouse & Whitworth, 2015). The advantages of survival analysis have been demonstrated in several longitudinal studies (Lee et al., 2015; Ng et al., 2011). This method allows censored observation, such as loss to follow‐up without failure, and the data with various observation times to be taken into account. The specific follow‐up times for each case are contributed to the analysis, making survival probability estimates more precise (Clark et al., 2003). Currently, the present study is the only report that demonstrates the possibility of the presence of radiographic pathology on the long‐term survival of primary teeth after a pulpectomy. Moreover, this was a retrospective longitudinal study with a large sample size, which allowed us to evaluate the association between prognostic factors and the survival of treated teeth. In this study, patient‐ and tooth‐related characteristics were collected from the GA and LA settings. The distributions of most characteristics were not significantly different between groups, except for age and type of root canal filling. The teeth in GA group were filled with Vitapex significantly more often than those in the LA group. This may be due to the difference in the handling and obturation technique between the two materials. ZOE is manually mixed and carried into the canals using a lentulo spiral drill. In contrast, Vitapex is a ready‐to‐use paste in a syringe, and the paste can be easily injected into the canals. Filling root canals with Vitapex requires fewer steps, is faster, and more convenient compared with ZOE, which could reduce the operation time in the GA group. Therefore, Vitapex tended to be used often in the GA compared with the LA group.

Traditionally, pulp‐treated teeth that presented signs of pathologic resorption or bone rarefaction in postoperative radiographs were classified by researchers as failures (Waterhouse & Whitworth, 2015), regardless of the clinical signs or the extent of pathology. However, according to Payne et al., a small degree of pathologic root resorption or radiolucency observed in primary teeth after pulpectomy was acceptable for clinicians, if clinical signs and symptoms were absent (Payne et al., 1993). Rather than the immediate extraction or retreatment of these teeth, practitioners usually decided to observe the affected teeth in the oral cavity for further evaluation at the next recall visit and the parents were advised to call the dentist if they developed any symptoms. This treatment option was more satisfying for the caregivers, because it retains the asymptomatic teeth in function and requires no further treatment at that time. Therefore, the cut‐off point for survival analysis in our study was set as the extraction or retreatment of the treated teeth, because this outcome explicitly represented pulpectomy primary tooth survival to the clinicians and caregivers.

Although pulpectomy is the recommended treatment for teeth with infected pulp tissue (American Academy of Pediatric Dentistry, 2019), some dentists prefer extracting pulpally involved primary teeth rather than performing endodontic treatment (Bowen et al., 2012). This might be due to the attitude of the dentists toward pulpectomy treatment. According to a survey on pulp treatment taught in U.S. dental schools, only 85% taught or performed pulpectomy on primary teeth (Dunston & Coll, 2008). Many dentists may be unfamiliar with endodontic treatment in primary teeth and may feel that extraction is less technique‐sensitive and more predictable compared with a pulpectomy, especially when delivering treatment under GA.

Repeated GA, resulting from previous treatment failure, is one of its most unfavorable consequences. Treatment under GA cannot be considered an operation without risk. The complications of GA range from immediate postoperative problems, such as nausea, vomiting, agitation, or a sore throat that resolves in several days, to life‐threatening issues, such as respiratory distress, laryngospasm, and cardiac arrest (Gonzalez et al., 2012; Needleman et al., 2008). Moreover, pulpectomy is known to be a more time‐consuming procedure, compared with extraction, which could affect the overall operation time. Time is also an important consideration for performing treatment under GA. The association of prolonged time under GA and the risk of post‐op complications and delayed recovery have been reported (Misal et al., 2016; Needleman et al., 2008). Moreover, increased overall GA time also decreases the efficiency of operation room utilization, increases waiting time for treatment under GA, and places a burden on health care system resources (Forsyth et al., 2012). Therefore, pulpectomy is not included in comprehensive dental treatment in some settings (El Batawi, 2014). The present study demonstrated the high survival rates of performing a pulpectomy in primary teeth under either GA or LA, suggesting that a pulpectomy provides a favorable outcome in either setting.

Currently, there are few studies that reported the survival of teeth receiving a pulpectomy under GA. Tang and Xu demonstrated that in 192 molars treated with Vitapex pulpectomy, the tooth survival rate was 79.12% at an 18‐month follow‐up (Tang & Xu, 2017). Similarly, Amin et al. found that 75.7% of teeth survived and needed no further treatment after pulpectomy at a 3‐year follow‐up (Amin et al., 2016). However, the type of root canal filling material was not reported in their study. In the present study, the survival rate of primary teeth receiving pulpectomy under GA was higher compared with previous studies. Differences in study methods may explain the disparate findings.

One factor that might has improved treatment outcome was that we used an EAL for working length determination. In our study, the working length of the treated tooth was maintained 1 mm short of the “Apex” reading on the apex locator display as described by Angwaravong and Panitvisai (Angwaravong & Panitvisai, 2009). EAL is claimed to be the most accurate method in working length determination in vivo, compared with radiographs and the tactile method. EAL demonstrated the least deviation in mean root length from the actual root length. In teeth without root resorption, EAL and radiographs demonstrated no significant difference in root length compared with the actual root length. In contrast, tactile sensation demonstrated a significant difference in teeth with and without root resorption (Wankhade et al., 2013). Using an EAL is claimed to be safe, convenient, and minimizes the child's radiation exposure. EAL use helps reduce the pulpectomy operation time (Ahmed, 2013). Moreover, this modality helps prevent over‐instrumentation and overfilling of the obturation material, which increases the risk of treatment failure or damaging the periapical tissue and developing tooth bud (Ahmed, 2013; Coll & Sadrian, 1996). Although data from a systematic review with meta‐analyses revealed that the pooled pulpectomy success was not significantly different between studies that used EAL or radiographs (Coll et al., 2020), no report directly compared pulpectomy success between different working length determination methods. Therefore, using an EAL during a pulpectomy under GA may be beneficial, because it may increase the survival rate of pulpectomized teeth and decrease the operation time under GA.

In this study, the survival rate of the teeth treated under GA was not significantly different from those treated under LA. Moreover, tooth survival following pulpectomy was not influenced by sex, age, tooth type, dental arch, molar type or location, root canal filling material type, final restoration type, or presence of pathologic root resorption. This study agreed with a previous study that found that tooth survival was not affected by these factors (Rawson et al., 2019). We found that a radiolucency at the periapical tissue or furcation area in the preoperative radiographs was the only factor significantly associated with 5‐year tooth survival following pulpectomy. Teeth with a preoperative radiolucency were prone to be extracted or retreated 3.9‐fold more often than teeth with less severe or no pathology on preoperative radiographs. We categorized the radiographic findings in three groups: (1) no pathology, (2) widened periodontal space and/or discontinuous lamina dura, and (3) radiolucency at the periapical area or furcation, to depict the extent of apical tissue destruction, which also reflect the stages and severity of the apical periodontitis. A radiolucency at the furcation or periapical tissue represents the most severe form of radiographic pathology, compared with teeth with either a widened PDL space/discontinuous lamina dura or no pathologic change. This radiolucency represents bone destruction, which is a hallmark of chronic apical periodontitis (Lin & Huang, 2015). Therefore, the infection at these teeth has progressed further in both time and extent, compared with teeth without a radiolucency, which might impede periradicular tissue healing after a pulpectomy and eventually contribute to tooth extraction.

Our results are consistent with the findings of Trairatvorakul and Chunlasikaiwan (Trairatvorakul & Chunlasikaiwan, 2008). They found that the molars that failed clinically or radiographically in the ZOE and Vitapex groups at a 12‐month follow‐up had a preoperative radiolucency. This finding supports the potential of a preoperative radiolucency as a prognostic variable affecting tooth survival following a pulpectomy. Therefore, this factor should be considered when selecting the best treatment choice for severely decayed primary teeth. Dentists should be aware that teeth with a preoperative radiolucency are more likely to fail compared with those without radiographic pathology. The survival probability of a tooth after treatment should be carefully discussed with caregivers prior to treatment, especially with uncooperative children for who repeated aggressive treatment might be difficult. The cost‐effectiveness between a pulpectomy and extracting these teeth should also be determined, especially in children who require treatment under GA. In these cases, extracting severely infected teeth may be preferred to a pulpectomy.

In addition to pathologic bone changes on a preoperative radiograph, preoperative root resorption has been used to predict the success of teeth that received a pulpectomy (Coll & Sadrian, 1996). A study found that teeth with excessive root resorption were more likely to fail compared with those without root resorption. This is because the radiographic changes in teeth with apical periodontitis can also present as external root resorption (Lin & Huang, 2015). In the present study, however, external pathologic root resorption was not significantly associated with tooth survival. This might be due to the small sample size in this subgroup, which could make the effect of this factor on tooth survival unclear. Therefore, studies with a larger sample size in this subgroup are required to confirm the association between this factor and pulpectomized tooth survival.

There are some limitations of our study. Because this was a retrospective study, some confounding factors could not be controlled, for example, more than one operator performed the pulpectomies. Other potential factors were not evaluated in this study, including the degree of physiologic root resorption, type of irrigant used, and pre‐operative pulp status. Moreover, the evaluation of the factors associated with primary tooth survival following a pulpectomy was compromised by the low event rate and low number of teeth in some variables, which may conceal the effect of a potential factor on tooth survival.

## CONFLICT OF INTEREST

There are no conflicts of interest to declare.

## AUTHOR CONTRIBUTIONS

Defined the research objective and collaborated with PA and OC in experimental design: Siriporn Songsiripradubboon. Contributing in data collection, analysis and results interpretation: Methaphon Songvejkasem. The manuscript co‐writing: Methaphon Songvejkasem, Prim Auychai, Oitip Chankanka, and Siriporn Songsiripradubboon.

## Data Availability

The data that support the findings of this study are available on request from the corresponding author. The data are not publicly available due to privacy or ethical restrictions.
